# Death of Dr. Beaumont

**Published:** 1853-06

**Authors:** 


### DEATH OF DR. BEAUMONT.

Dr. William Beaumont, known universally for his experiments on digestion, died at St. Louis, on the 25th of last April, in the 68th year of his age. He was born in Connecticut in the year 1785, and for nearly one third of his life was a member of the Medical Staff of the Army of the U. S. He is described by those who enjoyed his personal acquaintance as a man of noble moral qualities. On the occasion of his death resolutions respectful to his memory were adopted by the medical profession in St. Louis. The circumstance to which Dr. Beaumont owes his wide celebrity is the wound in the abdomen of his patient, Alexis St. Martin, which enabled him to perform a most interesting and instructive series of experiments in reference to the process of digestion.

This young man, a Canadian 18 years of age, recieved a charge of buck-shot in his left side, which carried away the integuments and muscles, fracturing some of the ribs, lacerating the diaphragm and lungs, and perforating the stomach. The food which he had taken at breakfast was escaping through the aperture when Dr. Beaumont saw him half an hour after the accident. The wound was received on the 6th of June, 1822, and in a year, after exfoliation of ribs, many abscesses, and great suffering, the extensive injury was nearly repaired, but a perforation leading into the stomach remained, two inches and a half in circumference. His food escaped from the stomach by this aperture for some time, but eventually a fold of the inner coats of the organ formed a valve which remedied this evil, but the valvular formation was easily depressed by the finger, allowing the food to be extracted at pleasure. He became, in the end, active, athletic, and hearty, indulging his appetite like persons in perfect health. Dr. Beaumont instituted a great number o experiments on the function of his stomach thus laid open to view, which every writer on physiology since that time has noticed. At a later period, Prof. Dunglison repeated many of these experiments, and the results are given in his works on Physiology and Hygiene. From these observations on this singular case may be said to date our precise knowledge in regard to the nature of digestion. The researches of Dr. Beaumont were conducted with judgment and care, and form a body of facts which will always hold their place in physiological science, and connect with it his name as a fortunate investigator.

